# The Eye Tooth and the Eye

**Published:** 1875-05

**Authors:** Jas. Campbell

**Affiliations:** St. Louis.


### ARTICLE VI.

### *The Eye Tooth and the Eye.*

BY DR. JAS. CAMPBELL, ST. LOUIS.

Read before a Physicians' Club.

In all popular legends or common beliefs we may generally find a germ of truth, if they are examined carefully.

It is a fact, fixed in the minds of most people, that there is a direct connection between the "eye tooth" and the sense of vision. That with the loss or injury of this tooth, there is a corresponding injury to the sight. This is one of the popular beliefs, and one upon which little has been said by way of explanation.

It is difficult for the anatomist to trace out, or the physiologist to find any direct connection between the nerve of special sense that gives vision, and the ordinary sensitive branch of the trifacial which supplies the teeth. Yet many facts connecting the two seem to argue well for the popular belief.

Cases where irritation of disease of the canines is accompanied by deterioration, and sometimes even temporary total loss of sight, are by no means rare. All of us are cognizant of such cases. In our journals they are constantly appearing or reappearing as phenomenal and strange, and related as cases of interest. And the connection is not accidental, but the relations of cause or effect are so well established and admitted that we need not stop to consider it here.

Let us briefly examine the facts so established. It is the every day observation of the dentist to find pain in the lower diseased teeth, associated with severe pain in the upper and sound teeth of the same side. When we consider the manner of distribution of the great trifacial, we can see no inconsistency in explaining this by "reflex-action," though the filaments of this most sensitive of all the nerves. And it is convenient to dismiss it thus. But the gap between the eye, that is its visual function, and the sensitive nerves supplying the teeth is wider, and we cannot so conveniently step across.

We are taught to believe that each system of nerves has its own special function. That light affects only the optic nerve; sound the auditory; sensation the sensitive, etc.; but the connection between the irritation of the sensitive dental branch of the fifth pair and the eye remains as a fact, and we must examine their relations as best we can.

. It is true that the first or ophthalmic branch of the trifacial is distributed to the eye and some of the surrounding tissues; and the same theory of reflex-action, which associated and explained the connection of the upper sound teeth with the lower and diseased teeth, might, with equal reason, be applied to explain the connection between the first and second branch of the trifacial, as between the second and third branch. And were the connections in the cases related, those of associated pain or irritation, it would be easy to so dispose of it, and cases of this kind are constantly recurring; and many dismiss the subject thus, without further thought. But unfortunately for this theory, the ophthalmic division of the fifth nerve has little, if any direct connection with the sense of vision, it merely supplying the parts to which it is distributed with ordinary sensibility. The reverse was until recently advocated; for experimenters and authorities on the subject found that division of the fifth nerve or its ophthalmic branch was followed by injury to the sight and a diseased condition of the cornea; hence it was supposed the fifth nerve, in addition to its sensitive office, must have a direct influence upon the nutrition of the eye, as also upon its functional activity. But more careful observers found that when the division of the fifth was made behind the point where the sympathetic joins with it (on a level with the casserion ganglion,) vision was not affected. And the experiments of Snellen and others have conclusively proven that the corneal ulceration and degeneration of the parts which follow the division or paralysis of the fifth nerve depend, not directly, but indirectly upon that fact, and that it may be prevented, if the eye is externally protected from external irritations; that is owing to the insensibility of the parts which follows division of the fifth; the faithful sentinel sensation being gone it does not feel, and hence fails to resent external irritation, which soon causes the changes in the corneal tissues.

But in the cases related of failure of sight, depending upon diseased teeth, the external eye is reported as presenting no evidence of abnormal change; and there is little or no pain accompanying the gradual fading of sight. Of necessity the connection must be through nerves, for without nerves there is no activity. From our brief examination of the fifth nerve we see that it is necessary to look to another channel through which the influence is transmitted. And thus we turn to the great sympathetic for a moment.

The sympathetic, owing to natural obstacles which stand in the way of experiment, is comparatively little understood, but we know it to be the great nutritive system, and that its filaments accompany and have much to do with regulating arterial activity. The fifth pair is connected, in different parts of its course, with a number of small ganglia of the sympathetic. The ophthalmic ganglion receives a sensitive root from the opthalmic division of the fifth. A small sympathetic filament from the ophthalmic ganglion penetrates the optic nerve with the arteria-centralis retinæ, which supplies the retina. Now as division of the fifth nerve affects the sight only when it includes fibres of the sympathetic, it would seem reasonable to suppose that it is through the sympathetic that the sight becomes involved.

The conditions necessary to vision, unobstructed dioptric media (cornea, lens, etc.,) through which external impressions may pass; a normal condition of the retinal nervous expansion to receive such impressions; ability of the optic to transmit the impressions so received; and finally condition of the sensorium to recognize impressions so conducted,—and thus explanation of any variation in sight must be sought in a defect in any one or more of these links. In the cases under consideration, the dioptric media are reported as unaffected, nor can we think that it is the sensorium at fault; hence we may narrow the affected parts to the optic nerve and its retinal expansion.

We have now reached a point where reasons are in order: but although we have traced the subject this far, almost to warrant a rational conclusion, just here a great obstacle stands in the way, which prevents, for the present, a more definite answer, that is, in none of the cases presented has a careful ophthalmoscopic examination of the inner eye been reported. This is of vital importance and would decide many things. Without it no one versed on the eye would attempt to decide so important a question.

Experiment proves that a division of the sympathetic fibres causes vascular congestion of the parts to which they are distributed, which may last for several weeks; and also that direct irritation of these fibres causes the reverse effect. Either of these conditions would affect the sight. Is it then owing to over-vascularity of the optic nerve and retina, as would be caused by *division* of the ophthalmic, or the reverse condition like that caused by *irritation* of its fibres.

We may ask is this connection possible? Without at doubt it is, and can be verified by analogous nervous reflex-action in other parts of the body. Disgusting sights or odors may cause nausea; may hasten or retard menstrual flow; may induce premature delivery. That is we have many evidences of reflex-action from sensitive points through the cerebro-spinal and the sympathetic system to the different organs and distant parts of the body. Thus the path of transmission is clear from the eye to other points and organs of the body; and for a like reason the reverse must be so.

The sight may be affected by many and varied causes, some clear and others obscure. And quite an exceptional degree of diagnostic skill is requisite to differentially distinguish many of its variations. And hence when the competent oculist has searched in vain for a hidden cause let him not forget to examine the teeth. And the dentist should also remember that it is very possible for diseased teeth to co-exist with impaired vision, with not the slightest connection between them; that this peculiar state is indeed quite exceptional, and occurs in only a limited number of cases.—*Missouri Dental Journal.*

---

